# Using a trait-based approach to optimize mixotrophic growth of the red microalga *Porphyridium purpureum* towards fatty acid production

**DOI:** 10.1186/s13068-018-1277-7

**Published:** 2018-10-04

**Authors:** Kailin Jiao, Wupeng Xiao, Yuanchao Xu, Xianhai Zeng, Shih-Hsin Ho, Edward A. Laws, Yinghua Lu, Xueping Ling, Tuo Shi, Yong Sun, Xing Tang, Lu Lin

**Affiliations:** 1 College of Energy, Xiamen University, Xiamen, 361102 People’s Republic of China; 2 State Key Laboratory of Marine Environmental Science/Fujian Provincial Key Laboratory for Coastal Ecology and Environmental Studies, Xiamen University, Xiamen, 361102 China; 3 Shangdong Provincial Key Lab. of Microbial Engineering, Qilu University of Technology (Shandong Academy of Sciences), Jinan, 250353 Shangdong People’s Republic of China; 4 Fujian Engineering and Research Center of Clean and High-Valued Conversion Technology for Biomass, Xiamen Key Laboratory of Clean and High-valued Conversion Technology of Biomass, Xiamen University, Xiamen, 361102 China; 5 State Key Laboratory of Urban Water Resource and Environment, School of Environment, Harbin Institute of Technology, Harbin, 150006 China; 6 Department of Environmental Sciences, School of the Coast & Environment, Louisiana State University, Baton Rouge, LA 70803 USA; 7 Department of Chemical and Biochemical Engineering, College of Chemistry and Chemical Engineering, Xiamen University, Xiamen, 361005 China

**Keywords:** *Porphyridium purpureum*, Arachidonic acid, Organic carbon, Generalized additive models, Trait-based approach

## Abstract

**Background:**

Organic carbon sources have been reported to simultaneously increase the growth and lipid accumulation in microalgae. However, there have been no studies of the mixotrophic growth of *Porphyridium purpureum* in organic carbon media. In this study, three organic carbon sources, glucose, sodium acetate, and glycerol were used as substrates for the mixotrophic growth of *P. purpureum*. Moreover, a novel trait-based approach combined with Generalized Additive Modeling was conducted to determine the dosage of each organic carbon source that optimized the concentration of cell biomass or fatty acid.

**Results:**

A 0.50% (*w*/*v*) dosage of glucose was optimum for the enhancement of the cell growth of *P. purpureum*, whereas sodium acetate performed well in enhancing cell growth, arachidonic acid (ARA) and eicosapentaenoic acid (EPA) content, and glycerol was characterized by its best performance in promoting both cell growth and ARA/EPA ratio. The optimum dosages of sodium acetate and glycerol for the ARA concentration were 0.25% (*w*/*v*) and 0.38% (*v*/*v*), respectively. An ARA concentration of 211.47 mg L^−1^ was obtained at the optimum dosage of glycerol, which is the highest ever reported.

**Conclusions:**

The results suggested that a comprehensive consider of several traits offers an effective strategy to select an optimum dosage for economic and safe microalgae cultivation. This study represents the first attempt of mixotrophic growth of *P. purpureum* and proved that both biomass and ARA accumulation could be enhanced under supplements of organic carbon sources, which brightens the commercial cultivation of microalgae for ARA production.

## Background

Arachidonic acid (ARA, C20:4ω6) derived from microalgae is in high demand because ARA is one of the most abundant poly-unsaturated fatty acids (PUFAs) in the brain [1], and its traditional sources, including marine fish oil and animal tissues, are not sustainable and are becoming more and more scarce [2]. However, the fatty acid content of microalgae is very sensitive to culture conditions. The major PUFA that benefits from favorable growth conditions is eicosapentaenoic acid (EPA, C20:5ω3), which is the downstream product of ARA [3–7]. In contrast, a high ARA content is usually obtained under stressful conditions. These conditions include low light intensity, suboptimal temperature, suboptimal pH, high salinity, and micronutrient limitation [3–7]. All of these conditions are detrimental to the production of algal biomass. The result is an increase of ARA content but a decrease in ARA production, i.e., a decrease in ARA content × algal biomass production.

To overcome this problem, more and more studies have involved the addition of doses of organic carbon into the growth medium to exploit the ability of microalgae to grow in a mixotrophic mode. Simultaneous increases in growth and lipid accumulation have been reported for a variety of carbon sources [8–12]. However, no studies have addressed the effects of organic carbon substrates on ARA accumulation in microalgae. Determination of whether inexpensive carbon sources could enhance microalgal production of ARA is, therefore, needed.

The performance of microalgae in culture systems depends on their physiological condition, which is typically characterized in terms of their optimal irradiance, maximum rate of photosynthesis, and their ability to take up and utilize substrates (e.g., maximum uptake rates and half-saturation constants) [13]. Trade-offs among these traits result in fundamental niches of microalgae with respect to resources [14]. Fundamental niches also exhibit important characteristic traits, such as the mean and breadth of the niche [15, 16]. Consideration of all traits simultaneously is, therefore, important for the process of resource optimization. However, trait values can be estimated only when a specific response function can be obtained. Unfortunately, most of the relationships between microalgae and resources cannot be simulated with simple parametric models, because the relationships are generally nonlinear or not normally distributed [17]. Generalized additive modeling (GAM) allows for rather flexible specification of variables and has the advantage of being nonparametric regarding the statistical distribution of the data [18–21]. GAMs have been widely applied in environmental monitoring [22], ecology [16, 23, 24] and medicine [25] but have not been used to optimize the conditions for culturing microalgae.

The only microalga reported to produce ARA in significant quantities is the red unicellular rhodophyte *Porphyridium purpureum* [4, 26]. ARA can account for as much as 40% of total fatty acids (TFA), but only under stressful conditions [4–7]. To date, there have been no studies of the mixotrophic growth of *P. purpureum* in organic carbon media. In this study, three organic carbon sources, glucose, sodium acetate, and glycerol [11, 12, 27, 28] were used as substrates for the mixotrophic growth of *P. purpureum*. We used GAM and a novel trait-based approach to determine the dosage of each organic carbon source that optimized the concentration of cell biomass or ARA.

## Methods

### Microalgae culture systems

The microalga *P*. *purpureum* CoE1 that was cultivated in this study was previously screened and has been maintained by the authors’ research group [7]. The experiments with glucose, sodium acetate, and glycerol were carried out at the same time. Experimental cultures were grown in 1-L flasks containing 500 mL of artificial seawater medium (ASW) [29]. All flasks were placed evenly in the same incubator under continuous illumination at a light intensity of 165 µmol photons m^−2^ s^−1^ with cool white fluorescent lamps. The temperature was maintained at 25 °C, and the cultures were aerated at a rate of 1 L min^−1^. The pH during the cultivation was adjusted to be 7.6 with Tris–HCl buffer every other day. The mixotrophic cultivations were operated by adding different dosages of glucose (*w*/*v*), sodium acetate (*w*/*v*), and glycerol (*v*/*v*). The dosages were all set to 0.05, 0.1, 0.25, 0.5, 0.75, or 1.0%. Preliminary experiments showed that this gradient was enough to capture the full range of the effects of these three carbon sources on algal biomass. An autotrophic group with no supplement of organic carbon sources was taken as the control group. The three series of experiments shared the same control group. All experiments were performed in triplicate. Cell biomass and organic carbon content were measured every other day. The cultivation lasted until the cell biomass was stable on the 18th day. The concentration and composition of fatty acids (FAs) were measured at the end of the cultivation.

### Measurement of algal cell biomass

The metric of microalgal biomass was the cell dry weight concentration (DW, g L^−1^), which was determined by measuring the optical density (OD_604nm_) of the cultures and an empirical relationship between DW and OD_604nm_ [7]. The dry weight was obtained by washing the cells twice with distilled water and drying them in an oven at 80 °C until a constant weight was achieved.

### Determination of organic carbon content

The content of glucose in the samples was determined using a dinitrosalicylic acid (DNS) method [30]. Acetate and glycerol contents were determined by gas chromatography according to Sundqvist et al. [31] and Wang et al. [32], respectively.

### Analyses of lipids and fatty acids

Lipid extraction for fatty acid analysis was conducted following the method of Bligh and Dyer [33]. In brief, about 0.1 g of lyophilized biomass of each sample was extracted with a chloroform–methanol–water solution. The lipid-containing chloroform phase in the substratum was dried to powder under a nitrogen stream. Fatty acid methyl esters (FAMEs) were prepared by esterification of the powdered lipids in a KOH–methanol solution with the C17:0 ester containing cyclohexane as the internal standard. The upper layer of the mixture was separated for FAME composition analysis.

The FAME composition analysis was conducted with a GC–MS system (ThermoFisher Trace 1300-ISQLT). The GC–MS system was equipped with an electron impact ionization detector and a TR-5MS column (30.0 m × 250 μm × 0.25 μm). The injection volume was 1 μL for each sample. The temperature of the injector and detector, the column flow rate, and the split ratio were 250 °C, 1.2 mL min^−1^, and 1:50, respectively. The running temperature was set as follows: 40 °C for 1 min, heating to 230 °C at 20 °C min^−1^, held at 230 °C for 1 min, heating to 270 °C at 3 °C min^−1^, and held at 270 °C for 2 min. An internal standard was used to quantify the weight (mg) of each fatty acid, and its cellular content (mg g^−1^) was calculated in terms of its weight per gram of algal powder.

### GAM modeling

GAM functions were constructed with a Gamma distribution to describe the functional relationship, *f*(*x*), between cell biomass or ARA concentration and dosage, *x*, using the function ‘*gam*’ in the R package ‘mgcv’ [19]. The use of Gamma distribution assured that the fitting values of the response variable (cell biomass or ARA concentration) were positive [19]. The model formulations were as follows:

1 $$f(x) = \alpha + s\left( x \right) + \varepsilon ,$$

where the symbol *α* is the intercept and *ε* the random noise. The term *s* indicates a one-dimensional nonlinear function based on thin plate regression splines. To avoid overfitting, according to Wood [19], the number of basis functions was constrained to be less than five, and each model effective degree of freedom was forced to count as 1.4 degrees of freedom in the generalized cross-validation (GCV) score. Similar treatments have been done in Liu et al. [34] and Chen et al. [35].

### Calculations of traits

If the GAM function, *f*(*x*), was unimodal, the maximum potential dosage of each carbon source for the cell biomass or ARA content, *p*_max_, was calculated according to the following equation:

2 $$f^{\prime}\left( {p_{{\text{max}}} } \right) = \mathop {\lim }\limits_{\Delta x \to 0} \frac{{f\left( {p_{{\text{max}}} + \Delta x} \right) - f(p_{{\text{max}}} )}}{\Delta x} = 0$$

The mean (*μ*) and standard deviation (*σ*) of the dosage were defined by

3 $$\mu = \frac{{\mathop \smallint \nolimits_{0}^{{t_{{\text{max}}} }} xf\left( x \right)\text{d}x}}{{\mathop \smallint \nolimits_{0}^{{t_{{\text{max}}} }} f\left( x \right)\text{d}x}}$$

4 $$\sigma^{2} = \frac{{\mathop \smallint \nolimits_{0}^{{t_{{\text{max}}} }} \left( {x - \mu } \right)^{2} f\left( x \right)\text{d}x }}{{\mathop \smallint \nolimits_{0}^{{t_{{\text{max}}} }} f\left( x \right)\text{d}x }}$$

In Eqs. (3) and (4), *t*_max_ is the least dosage that satisfies the condition *f(t*_max_) ≥ *f*(0). We call the trait *t*_max_ the maximum tolerance dosage. The parameters *μ* and σ represent the mean dosage of a carbon source and the range (breadth) of the dosage within which the cell biomass or ARA content is high [15, 16], and we interpret these two traits as a simple description of the mean safe dosage and breadth of the mean safe dosage, respectively.

### Other analyses

The fatty acid compositions and their cellular contents obtained with the three carbon sources were analyzed with a principal component analysis (PCA) using the R package ‘vegan’ [36]. To contrast the overall difference in the cell biomass between two dosages of a carbon source, a generalized linear mixed-effects model (GLMM) was used with the ‘lme4’ package for R [37]. Time was taken as the random effect. A Kruskal–Wallis test was used to analyze the overall difference in variances between dosages at the same sampling time [38]. All analyses in this study were done using the R version 3.4.4 (R Development Core Team 2018) [39].

## Results

### Effects of organic carbon sources on the cell growth of *P. purpureum*

**Fig. 1:**
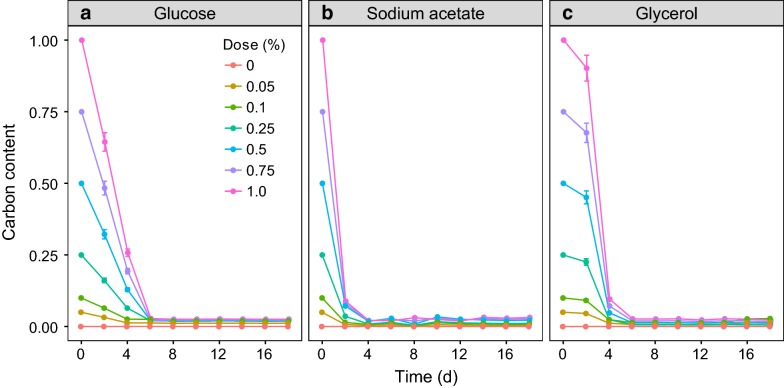
Changes in organic carbon sources along the cultivation course. **a** Glucose. **b** Sodium acetate. **c** Glycerol

**Fig. 2:**
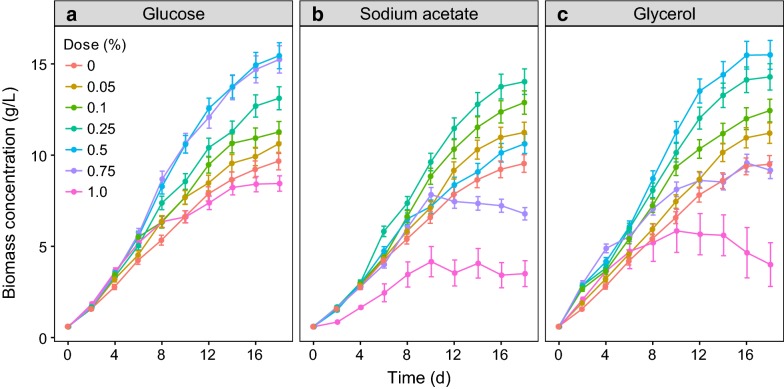
The cell biomass of *P. purpureum* in the presence of different organic carbon sources. **a** Glucose. **b** Sodium acetate. **c** Glycerol

**Fig. 3:**
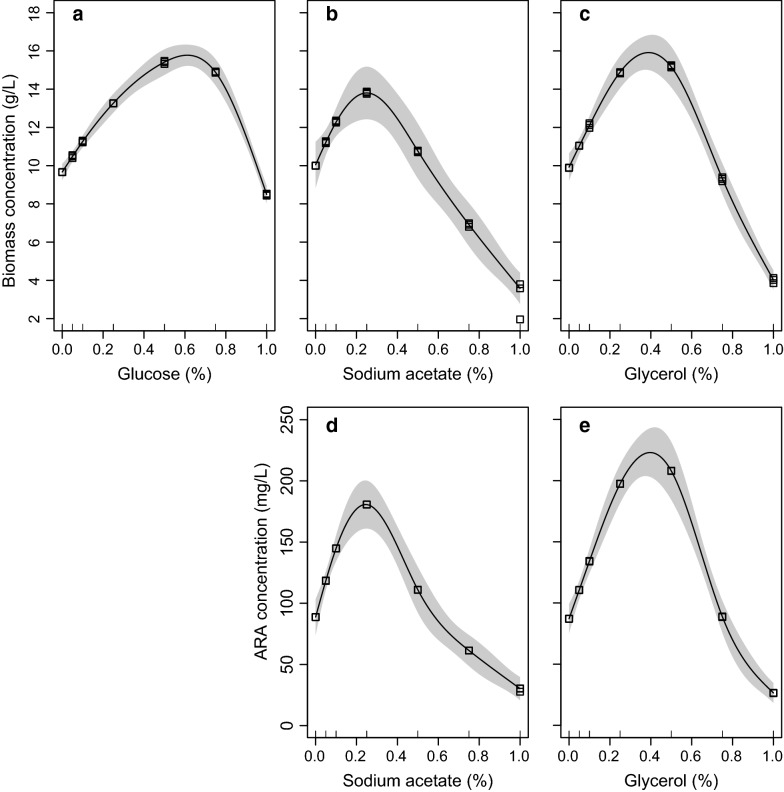
Effects of organic carbon sources on cell biomass and ARA concentration of *P. purpureum* using a GAM modeling. **a**–**c** The cell biomass versus **a** Glucose, **b** Sodium acetate, and **c** Glycerol. **d**, **e** The ARA concentration versus **d** Sodium acetate and **e** Glycerol. Shaded areas are 95% confidence intervals

**Fig. 4:**
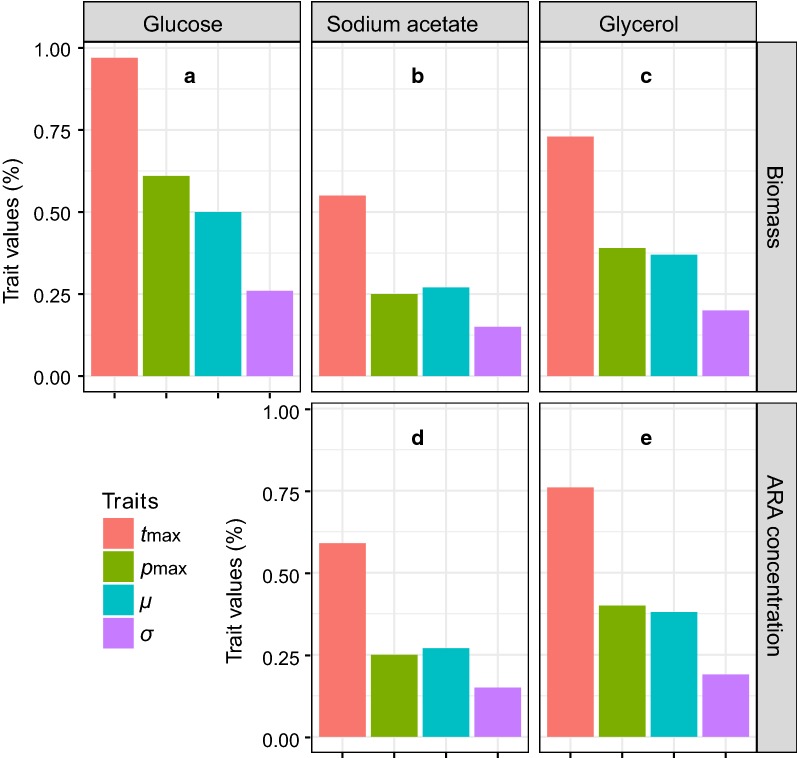
Trait values for cell biomass and ARA concentration of *P. purpureum*. The traits are *t*_max_, the maximum tolerance dosage, *p*_max_, the maximum potential dosage, *μ*, the mean safe dosage, and *σ*, the breadth of the mean safe dosage. **a**–**c** The trait values in terms of cell biomass versus **a** Glucose, **b** Sodium acetate, and **c** Glycerol. **d**, **e** The trait values in terms of ARA concentration versus **d** Sodium acetate and **e** Glycerol

The residual of glucose in the cultures dropped rapidly to about zero after 6 days (Fig. 1a). *P. purpureum* benefited from a supplement of glucose in the range of 0–0.75% but the 1.0% group was worse than the control after the tenth day (Fig. 2a). A significant increase in the biomass of *P. purpureum* versus the control occurred when the glucose concentration was more than 0.10% (GLMM, *p* < 0.05). The highest biomass, 15.36 g L^−1^, was achieved with the 0.50% group (Table 1), but there was no significant difference between the 0.50 and 0.75% groups throughout the time course (GLMM, *p* > 0.05) (Fig. 2a). The GAM revealed that the biomass on the 18th day showed a highly left-skewed unimodal relationship with doses of glucose (*R*^2^ = 0.95, *p* < 0.05, Fig. 3a). The GAM function estimated the maximum-potential-biomass dosage of glucose to be 0.61% (Fig. 4a). The validation experiment obtained a biomass of 15.85 g L^−1^ at that dosage (Table 2), which was slightly higher than the biomass obtained at a dosage of 0.50%, but there was no significant difference (*t* test, *p* > 0.05). The GAM also predicted that the maximum tolerance dosage for glucose was 0.97% (Fig. 4a). The mean safe dosage and the breadth of the mean safe dosage for glucose, calculated with Eqs. (3) and (4), were 0.50 and 0.26%, respectively (Fig. 4a).

**Table 1:** Final cell biomass and fatty acid production of *P. purpureum* supplemented with organic carbon sources at 18th day

Carbon source	Dose (%)	Biomass (g L^−1^)	Content (mg g^−1^)				Concentration (mg L^−1^)		
			ARA	EPA	TFA	ARA/EPA	ARA	EPA	TFA
Control	0.00	9.58 (0.48)	8.95 (0.17)	6.40 (0.22)	45.62 (2.82)	1.40 (0.05)	85.7 (1.0)	61.3 (2.1)	437.0 (25.8)
Glucose	0.05	10.54 (0.53)	9.10 (0.02)	6.88 (0.43)	47.00 (3.25)	1.32 (0.04)	95.9 (7.6)	72.5 (6.4)	495.4 (27.5)
	0.10	11.32 (0.57)	8.64 (0.83)	5.78 (0.82)	44.64 (2.32)	1.49 (0.07)	97.8 (4.9)	65.4 (7.3)	505.3 (25.7)
	0.25	13.17 (0.63)	8.86 (0.23)	6.12 (0.36)	46.49 (3.38)	1.45 (0.08)	116.7 (2.1)	80.6 (5.0)	612.3 (36.0)
	0.50	15.36 (0.71)	9.03 (0.57)	5.86 (0.39)	46.76 (2.61)	1.54 (0.03)	138.7 (2.1)	90.0 (5.4)	718.2 (39.5)
	0.75	15.04 (0.75)	8.48 (0.15)	6.63 (0.15)	45.22 (2.45)	1.28 (0.05)	127.5 (1.9)	99.7 (9.4)	680.1 (30.0)
	1.00	8.45 (0.42)	8.72 (0.15)	5.05 (0.52)	44.29 (3.76)	1.73 (0.05)	73.7 (0.1)	42.7 (3.3)	374.3 (17.8)
Sodium acetate	0.05	11.29 (0.52)	10.58 (1.11)	7.25 (0.63)	49.28 (4.64)	1.46 (0.07)	119.5 (18.6)	81.9 (8.4)	556.4 (25.8)
	0.10	12.73 (0.57)	11.66 (0.53)	7.61 (0.83)	51.84 (2.92)	1.53 (0.07)	148.4 (2.0)	96.9 (6.3)	659.9 (36.0)
	0.25	14.05 (0.70)	12.85 (0.03)	8.62 (0.34)	54.17 (2.85)	1.49 (0.06)	180.5 (10.4)	121.1 (7.4)	761.1 (40.1)
	0.50	10.55 (0.46)	10.34 (0.61)	7.91 (0.93)	45.15 (2.75)	1.31 (0.05)	109.1 (10.8)	83.5 (8.1)	476.3 (28.3)
	0.75	6.86 (0.37)	8.88 (0.61)	6.35 (0.75)	42.13 (3.65)	1.40 (0.05)	60.9 (1.3)	43.6 (4.1)	289.0 (15.4)
	1.00	3.54 (0.81)	8.24 (0.37)	5.57 (0.78)	38.16 (1.98)	1.48 (0.07)	29.2 (12.5)	19.7 (2.9)	135.1 (7.6)
Glycerol	0.05	11.27 (0.56)	9.75 (0.67)	5.61 (0.82)	50.80 (3.54)	1.74 (0.08)	109.9 (8.4)	63.2 (6.1)	572.5 (26.4)
	0.10	12.37 (0.62)	10.89 (0.92)	4.55 (0.27)	56.00 (2.88)	2.39 (0.10)	134.7 (28.1)	56.3 (2.2)	692.7 (36.4)
	0.25	14.38 (0.72)	13.43 (0.60)	4.37 (0.28)	62.22 (4.11)	3.07 (0.12)	193.1 (14.6)	62.8 (4.9)	894.7 (47.3)
	0.50	15.72 (0.79)	13.67 (0.84)	4.75 (0.57)	63.41 (4.17)	2.88 (0.17)	214.9 (26.8)	74.7 (7.3)	996.8 (39.8)
	0.75	9.13 (0.46)	9.44 (0.98)	3.98 (0.91)	58.56 (3.28)	2.37 (0.16)	86.2 (16.9)	36.3 (2.8)	534.7 (27.4)
	1.00	4.00 (1.2)	6.45 (0.13)	2.66 (0.33)	48.64 (3.42)	2.42 (0.14)	25.8 (3.8)	10.6 (1.5)	194.6 (12.7)

The values in parentheses represent standard deviations of three replicates

**Table 2:** Fitted (*f*) and measured (*m*) cell biomass and ARA concentration of *P. purpureum* at the maximum potential dosage (*p*_max_) and the mean safe dosage (*μ*)

	Biomass (g L^−1 ^± SD)			ARA concentration (mg L^−1 ^± SD)	
	Glucose	Sodium acetate	Glycerol	Sodium acetate	Glycerol
*f*(*p*_max_)	15.77 ± 0.32	13.76 ± 0.85	15.88 ± 0.56	178.0 ± 12.5	222.3 ± 12.6
*m*(*p*_max_)	15.85 ± 0.84	14.05 ± 0.70	16.11 ± 0.26	180.5 ± 10.4	224.3 ± 10.0
*f*(*µ*)	15.34 ± 0.36	13.73 ± 0.84	15.86 ± 0.55	177.7 ± 12.1	221.9 ± 12.0
*m*(*µ*)	15.36 ± 0.71	13.54 ± 1.08	16.06 ± 0.47	169.1 ± 15.0	211.5 ± 11.8

All parameters were tested in three replicates

The residual of sodium acetate declined sharply after the first 2 days and then dropped to very low concentrations after 4 days (Fig. 1b). The growth of *P. purpureum* was very sensitive to sodium acetate (Fig. 2b). A small dosage (0.05%) of sodium acetate resulted in a significant enhancement of cell biomass versus the control (GLMM, *p* < 0.05). This enhancement was maintained up to a dosage of 0.25%, above which no significant effect (0.5%) or negative effects (0.75 and 1.0%) were found (Fig. 2b). The highest cell biomass of 14.05 g L^−1^ was obtained at a dosage of 0.25% (Table 1). The GAM estimated a trend with a steep increase followed by a sharp decrease for the biomass obtained on the 18th day as the doses of sodium acetate increased (*R*^2^ = 0.95, *p* < 0.05, Fig. 3b). A dose of 0.25% sodium acetate was predicted to result in the maximum potential biomass (Fig. 4b). The maximum tolerance dosage, the mean safe dosage, and the breadth of the mean safe dosage for sodium acetate were 0.55, 0.27 and 0.15%, respectively (Fig. 4b). The experimental biomass at the mean safe dosage was 13.54 g L^−1^ (Table 2), which was slightly lower than the maximum biomass, but there was no significant difference (*t* test, *p* > 0.05).

When glycerol was used as the organic carbon source, its concentration also decreased to about zero after 6 days (Fig. 1c). The highest cell biomass of 15.72 g L^−1^ was obtained in the 0.5% group and was significantly higher than the cell biomass achieved with other tested groups (GLMM, *p* < 0.05) (Fig. 2c). Increasing the concentration to 1.0% reduced the biomass to a level significantly below the control (GLMM, *p* < 0.05) (Fig. 2c). The GAM estimated that the cell biomass on the 18th day increased with increasing small doses of glycerol, reached a maximum of 15.88 g L^−1^ at a dosage of 0.39%, and decreased at higher dosages (*R*^2^ = 0.95, *p* < 0.05, Fig. 3c, Table 2). The predicted maximum tolerance dosage of glycerol was 0.73%. The mean safe dosage and its breadth were estimated to be 0.37 and 0.20%, respectively (Fig. 4c). The experimental concentrations of cell biomass were 16.11 g L^−1^ and 16.06 g L^−1^ at the maximum-potential-biomass dosage and the mean safe dosage, respectively (Table 2). There was no significant difference between these biomass concentrations (*t* test, *p* > 0.05).

### Effects of organic carbon sources on fatty acids

The ranges of the ARA and TFA contents were 8.48–9.10 mg g^−1^ and 44.29–47.00 mg g^−1^, respectively (Table 1), in the glucose experimental groups, which were not significantly different from the control results (Kruskal–Wallis test, *p* > 0.05). The highest contents of ARA, EPA, and TFA in the sodium acetate treatments were 12.85 mg g^−1^, 8.62 mg g^−1^, and 54.17 mg g^−1^, respectively. All of these contents were obtained with the 0.25% dosage group and were 1.44, 1.35, and 1.19 times those of the control, respectively. The combination of high fatty acid content and high cell biomass resulted in concentrations of ARA, EPA, and TFA of 180.5 mg L^−1^, 121.1 mg L^−1^, and 761.1 mg L^−1^, respectively. The highest ARA and TFA contents in the glycerol treatments were 13.67 mg g^−1^ and 63.41 mg g^−1^, respectively. Both were obtained at a dosage of 0.5%, and the corresponding ARA and TFA concentrations were 214.9 mg L^−1^ and 996.8 mg L^−1^, respectively. However, the EPA content in the glycerol treatment was in the range 2.66–5.61 mg g^−1^, which was lower than the control content (6.4 mg g^−1^). The ARA/EPA ratios were significantly higher in the glycerol treatments than in the sodium acetate treatments (*t* test, *p* < 0.05).

The GAM revealed that the relationship between the ARA concentration of *P. purpureum* was unimodal and significant for sodium acetate (*R*^2^ = 0.96, *p* < 0.05, Fig. 2d) and glycerol (*R*^2^ = 0.93, *p* < 0.05, Fig. 3e). The maximum-potential-concentration dosage (0.25%), the mean safe dosage (0.27%), and the breadth of the mean safe dosage (0.15%) of sodium acetate were the same for the concentrations of ARA and cell biomass (Fig. 4d). The validation experiment produced an ARA concentration of 180.5 mg L^−1^ at the maximum-potential-concentration dosage and 169.1 mg L^−1^ at the mean safe dosage (Table 2). For glycerol, the GAM predicted that the maximum tolerance dosage, the maximum-potential-concentration dosage, the mean safe dosage, and the breadth of the mean safe dosage were 0.76, 0.40, 0.38, and 0.19%, respectively (Fig. 4e). The measured ARA concentration at the safe dosage (211.5 mg L^−1^) was not significantly different from the concentration at the maximum-potential-concentration dosage (224.3 mg L^−1^) (Table 2). Because ARA content remained stable among different doses of glucose, no effort was made to use the GAM model to describe the effect of glucose on the ARA concentration of *P. purpureum*.

**Fig. 5:**
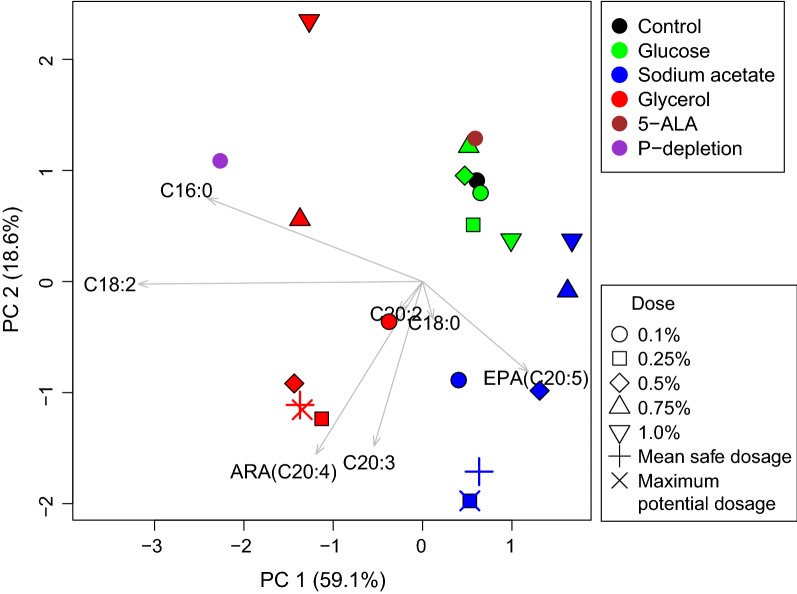
Principal component analysis based on fatty acid content of *P. purpureum* obtained from various conditions. The first two components represented 78% of the information of fatty acid compositions

The PCA of FA content revealed that the fatty acid composition of *P. purpureum* differed greatly between the three carbon sources (Fig. 5). The glucose treatment groups were ordered very close to the control, the indication being that glucose had no apparent effect on the fatty acid content of *P. purpureum*. All the sodium acetate treatments were located in the positive direction of EPA, and the maximum-potential-concentration dosage (0.25%) and the mean safe dosage (0.27%) were also located on the positive side of ARA. This result reflected the enhancement of both ARA and EPA production by sodium acetate. The glycerol treatments, which had positive effects on cell growth (0.1–0.5%), were near the positive direction of ARA, but not EPA. The indication is that glycerol is an accelerator unique for ARA accumulation in *P. purpureum*.

## Discussion

### Potent effects of organic carbon sources on cell biomass and ARA concentration of *P. purpureum*

As expected, appropriate doses of glucose, sodium acetate, and glycerol apparently enhanced the cell growth of *P. purpureum* (Fig. 2). This conclusion is consistent with the reported effects of these carbon sources on other microalgae, such as the effects of glucose and sodium acetate on *Monoraphidium* [12, 40–42] and *Chlorella* [28], and the effect of glycerol on *Chlorella* [43, 44]. Although the underlying mechanism remains to be determined, the results indicate that *P. purpureum* can grow better under mixotrophic conditions than under strictly autotrophic conditions, at least for the three tested organic carbon sources. The highest cell biomasses obtained from supplements of glucose, sodium acetate, and glycerol were 15.85 g L^−1^, 14.05 g L^−1^, 16.11 g L^−1^, respectively. These values were significantly higher than the control (9.58 g L^−1^) and approached the biomass (18.81 g L^−1^) obtained with an enhancement of phytohormones (5-ALA) [45].

Glucose had no apparent effect on the fatty acid content of *P. purpureum*, whereas sodium acetate enhanced the contents of both ARA and EPA, and glycerol produced the greatest enhancement of both ARA and the ARA/EPA ratio (Table 1 and Fig. 5). To assess the fatty acid composition associated with the three carbon sources, the previously published results obtained under conditions of phosphorous depletion (P-depletion) [7] and phytohormone (5-ALA) stimulation [45] were incorporated into the PCA analysis for comparison (Fig. 5). The phytohormone 5-ALA has been shown to greatly promote the growth of *P. purpureum* but to have no dramatic effect on its ARA content [45]; the opposite is true for the effect of P-depletion [7]. The PCA showed that the glucose treatments were ordered very close to the 5-ALA treatment, whereas the glycerol treatments were in the same direction on PC 1 as P-depletion (Fig. 5). These results suggest that mixotrophic growth of *P. purpureum* with an appropriate supplement of some organic carbon sources is an effective way to balance biomass and ARA content. Indeed, the highest ARA concentration obtained from a 0.40% dosage of glycerol (224.3 mg L^−1^) was significantly higher than that of both P-depletion (159.7 mg L^−1^) and 5-ALA promotion (170.4 mg L^−1^) conditions [7, 45]. Because ARA has not been produced via industrial cultivation of microalgae, the results of our study may greatly facilitate successful commercial cultivation of *P. purpureum* for ARA production in the future. However, further research is needed to identify the underlying molecular mechanisms that would enable changing the metabolic pathways of this alga and controlling the synthesis of its products.

### Trait-based approach to optimizing culture conditions

Unlike the parametric models used previously [17, 46], herein a rather flexible nonparametric model, GAM, was used to help optimize the doses of carbon sources in the medium. The effects of the three carbon sources on cell biomass and the ARA concentration of *P. purpureum* differed. The relationships could be quantified in terms of four traits: the maximum-potential-concentration dosage (*p*_max_), i.e., the dosage at which biomass or ARA concentration was maximum; the maximum tolerance dosage (*t*_max_), i.e., the dosage at which the biomass or ARA concentration was less than the control; the mean safe dosage (*μ*) within the range of tolerance dosage; and the breadth of the mean safe dosage (*σ*), i.e., the standard deviation of the mean safe dosage. This trait-based approach has been very popular in ecology in recent years [13, 15, 16]. A combination of this trait-based approach with GAM modeling was very effective in optimizing the microalgal culturing conditions.

Glucose had no effect on the ARA content, but its positive effect on cell biomass showed a highly left-skewed unimodal relationship, with a plateau around the maximum-potential-concentration dosage of 0.61%, and a sharply decreasing trend at higher dosages (Fig. 3d). The mean safe dosage should, therefore, be smaller than the maximum-potential-concentration dosage. Indeed, the mean safe dosage was estimated to be 0.50%. However, both the breadth of the mean safe dosage (0.26%) and the maximum tolerance dosage (0.97%) for glucose were the highest among the three carbon sources. Because the breadth of the mean safe dosage represents the dosage sensitivity [15, 16], this result indicated that the growth of *P. purpureum* was less sensitive to glucose. For large-scale cultivation of *P. purpureum* aimed at harvesting biomass, glucose might be preferred because it is relatively cheap, effective, and safe, but a mean safe dosage instead of a maximum-potential-concentration dosage is highly recommended.

The scenario for sodium acetate was different. The peak concentration of cell biomass was confined to a relatively narrow range of sodium acetate concentrations (Fig. 3b). Therefore, both the maximum tolerance dosage (0.55%) and the breadth of the safe dosage (0.15%) were the lowest among the three carbon sources (Fig. 4a–c). However, the response function was slightly right-skewed within the range of tolerance dosage (Fig. 3b), the result being that the mean safe dosage (0.27%) was slightly higher than the maximum tolerance dosage (0.25%) (Fig. 4b). The same was true for the ARA concentration (Fig. 3d). Therefore, for large-scale cultivation of *P. purpureum* aimed at harvesting biomass as well as ARA, sodium acetate would be a good candidate because maximum enhancement occurs at a small dosage, and this dosage is safe.

Both the cell biomass and ARA concentration of *P. purpureum* increased monotonically with increasing glycerol concentration before reaching a peak at the maximum-potential-concentration dosages and then decreased sharply. The relationships displayed slightly left-skewed shapes (Fig. 3c, e). Consequently, all the trait values were intermediate between those of the other two carbon sources (Fig. 4). The mean safe dosage of glycerol for ARA concentration (0.38%) was slightly lower than the maximum-potential-concentration dosage (0.40%) (Fig. 4e), but the corresponding ARA concentrations were not significantly different (Table 2). The mean safe dosage for cell biomass and its breadth were 0.37 and 0.20%, respectively (Fig. 4c), the indication being that the mean safe dosage for ARA concentration was also within the safe range for cell biomass. The fact that glycerol performed well in enhancing both cell biomass and ARA content at a moderate dosage suggests that a mean safe dosage of this carbon source is perfect for cultivation of *P. purpureum*.

## Conclusion

This study was a first step toward exploring the mixotrophic growth of *P. purpureum* and used supplements of three organic carbon sources—glucose, sodium acetate, and glycerol—to enhance ARA concentration. Also, a trait-based approach combined with a modern statistical model, GAM, was used to optimize the culturing conditions. The maximum ARA concentrations were 180.5 mg L^−1^ and 224.3 mg L^−1^ for a 0.25% dosage of sodium acetate and a 0.40% dosage of glycerol, respectively. The latter concentration is the highest ever reported. In addition to the maximum-potential-concentration dosage, the maximum tolerance dosage, mean safe dosage, and the breadth of the mean safe dosage were estimated for both cell biomass and ARA concentration of each carbon source. These traits were found to differ among the three tested carbon sources. The mean safe dosages were not always equal to the maximum potential dosages, but there were no significant differences between the corresponding cell biomass andARA concentrations. The results showed that a 0.5% dosage of glucose was relatively effective and safe for the enhancement of cell growth of *P. purpureum*. Sodium acetate did the best job of safely enhancing cell growth and the content of ARA and EPA at a small dosage (0.25%). Glycerol was distinguished by its unique effect of promoting both cell growth and the ARA/EPA ratio at a moderate dosage (0.38–0.40%). The results of this study suggest that a comprehensive consideration of traits facilitates selection of an economic and safe dosage for microalgae cultivation. The concept of a trait-based approach combined with GAM is a convenient way to optimize other culture conditions and energy processes, and thus represents a significant advancement of bioengineering technology. However, the mechanisms associated with *P. purpureum* uptake of organic carbon sources are still unclear, and elucidation thereof will require further study.

## Abbreviations

PUFA: poly-unsaturated fatty acids
ARA: arachidonic acid
EPA: eicosapentaenoic acid
TFA: total fatty acid
GAM: generalized additive modeling
ASW: artificial seawater medium
DW: dry weight
OD: optical density
DNS: dinitrosalicylic acid
FAMEs: fatty acid methyl esters
*p*_max_: the maximum potential dosage
*μ*: the mean dosage
*σ*: the breadth of the mean dosage
*t*_max_: the maximum tolerance dosage
PCA: principal component analysis
GLMM: generalized linear mixed-effects model

## References

[1] Crawford MA, Sinclair AJ. Nutritional influences in the evolution of mammalian brain. In: lipids, malnutrition and the developing brain. Ciba Foundation symposium. 1971:267–292. 10.1002/9780470719862.ch16 4949878

[2] Gill I, Valivety R (1997). Polyunsaturated fatty acids, part 1: occurrence, biological activities and applications. Trends Biotechnol.

[3] Ahern TJ, Katoh S, Sada E (1983). Arachidonic acid production by the red alga *Porphyridium cruentum*. Biotechnol Bioeng.

[4] Bigogno C, Khozin-Goldberg I, Boussiba S, Vonshak A, Cohen Z (2002). Lipid and fatty acid composition of the green oleaginous alga *Parietochloris incisa*, the richest plant source of arachidonic acid. Phytochemistry.

[5] Cohen Z, Vonshak A, Richmond A (2010). Effect of environmental conditions on fatty acid composition of the red alga *Porphyridium cruentum*: correlation to growth rate. J Phycol.

[6] Su G, Jiao K, Chang J, Li Z, Guo X, Sun Y, Zeng X, Lu Y, Lin L (2016). Enhancing total fatty acids and arachidonic acid production by the red microalgae *Porphyridium purpureum*. Bioresour Bioprocess.

[7] Su G, Jiao K, Li Z, Guo X, Chang J, Ndikubwimana T, Sun Y, Zeng X, Lu Y, Lin L (2016). Phosphate limitation promotes unsaturated fatty acids and arachidonic acid biosynthesis by microalgae *Porphyridium purpureum*. Bioprocess Biosyst Eng.

[8] Chu WL, Phang SM, Goh SH (1995). Influence of carbon source on growth, biochemical composition and pigmentation of *Ankistrodesmus convolutus*. J Appl Phycol.

[9] Chu WL, Phang SM, Goh SH (1996). Environmental effects on growth and biochemical composition of *Nitzschia inconspicua* Grunow. J Appl Phycol.

[10] Hu H, Gao K (2003). Optimization of growth and fatty acid composition of a unicellular marine picoplankton, *Nannochloropsis* sp., with enriched carbon sources. Biotech Lett.

[11] O’Grady J, Morgan JA (2011). Heterotrophic growth and lipid production of *Chlorella protothecoides* on glycerol. Bioprocess Biosyst Eng.

[12] Yee W (2015). Feasibility of various carbon sources and plant materials in enhancing the growth and biomass productivity of the freshwater microalgae *Monoraphidium griffithii* NS16. Biores Technol.

[13] Litchman E, Klausmeier CA (2008). Trait-based community ecology of phytoplankton. Annu Rev Ecol Evol Syst.

[14] Edwards KF, Litchman E, Klausmeier CA (2013). Functional traits explain phytoplankton community structure and seasonal dynamics in a marine ecosystem. Ecol Lett.

[15] Irwin AJ, Nelles AM, Finkel ZV (2012). Phytoplankton niches estimated from field data. Limnol Oceanogr.

[16] Xiao W, Wang L, Laws E, Xie Y, Chen J, Liu X, Chen B, Huang B (2018). Realized niches explain spatial gradients in seasonal abundance of phytoplankton groups in the South China Sea. Prog Oceanogr.

[17] Guihéneuf F, Stengel DB (2015). Towards the biorefinery concept: interaction of light, temperature and nitrogen for optimizing the co-production of high-value compounds in *Porphyridium purpureum*. Algal Res.

[18] Hastie TJ, Tibshirani RJ (1990). Generalized additive models.

[19] Wood S (2006). Generalized additive models: an introduction with R.

[20] Zuur AF, Ieno EN, Smith GM (2007). Analysing ecological data.

[21] Zuur AF (2009). Mixed effects models and extensions in ecology with R.

[22] Richards R, Tomlinson RB, Chaloupka M. Using generalized additive models to assess, explore and unify environmental monitoring datasets. In: Modelling for environment’s sake: proceedings of the 5th biennial conference of the international environmental modelling and software society, iEMSs 2010; 2010, p. 1412–1420.

[23] Boyce DG, Lewis MR, Worm B (2010). Global phytoplankton decline over the past century. Nature.

[24] Xiao W, Liu X, Irwin AJ, Laws EA, Wang L, Chen B, Zeng Y, Huang B (2018). Warming and eutrophication combine to restructure diatoms and dinoflagellates. Water Res.

[25] Barker A, Kamar J, Morton A, Berlowitz D (2009). Bridging the gap between research and practice: review of a targeted hospital inpatient fall prevention programme. Qual Saf Health Care.

[26] Cohen Z (1990). The production potential of eicosapentaenoic and arachidonic acids by the red alga *Porphyridium cruentum*. J Am Oil Chem Soc.

[27] Azma M, Mohamed MS, Mohamad R, Rahim RA, Ariff AB (2011). Improvement of medium composition for heterotrophic cultivation of green microalgae, *Tetraselmis suecica*, using response surface methodology. Biochem Eng J.

[28] Huang A, Sun L, Wu S, Liu C, Zhao P, Xie X, Wang G (2016). Utilization of glucose and acetate by *Chlorella* and the effect of multiple factors on cell composition. J Appl Phycol.

[29] Jones RF, Speer HL, Kury W (1963). Studies on the growth of the red alga *Porphyridium cruentum*. Physiol Plant.

[30] Miller GL (1959). Use of dinitrosalicylic acid reagent for determination of reducing sugar. Anal Biochem.

[31] Sundqvist B, Karlsson O, Westermark U (2006). Determination of formic-acid and acetic acid concentrations formed during hydrothermal treatment of birch wood and its relation to colour, strength and hardness. Wood Sci Technol.

[32] Wang L, Qian J, Hu Z, Zheng Y, Hu W (2006). Determination of dihydroxyacetone and glycerol in fermentation broth by pyrolytic methylation/gas chromatography. Anal Chim Acta.

[33] Bligh ELG, Dyer WJA (1959). A rapid method of total lipid extraction and purification. Can J Biochem Physiol.

[34] Liu X, Xiao W, Landry MR, Chiang KP, Wang L, Huang B (2016). Responses of phytoplankton communities to environmental variability in the East China Sea. Ecosystems.

[35] Chen B, Liu H, Huang B (2012). Environmental controlling mechanisms on bacterial abundance in the South China Sea inferred from generalized additive models (GAMs). J Sea Res.

[36] Borcard D, Gillet F, Legendre P (2011). Numerical ecology with R.

[37] Bates D, Mächler M, Bolker B, Walker S (2015). Fitting linear mixed-effects models using lme4. Stat Comput.

[38] Hollander M, Wolfe DA (1973). Non-parametric statistical methods.

[39] R Development Core Team. R: a language and environment for statistical computing. Vienna, Austria: R Foundation for Statistical Computing. 2018. Open access available at: http://cran.r-project.org.

[40] Yu X, Zhao P, He C, Li J, Tang X, Zhou J, Huang Z (2012). Isolation of a novel strain of *Monoraphidium* sp. and characterization of its potential application as biodiesel feedstock. Bioresour Technol.

[41] Patidar SK, Mitra M, George B, Soundarya R, Mishra S (2014). Potential of *Monoraphidium minutum* for carbon sequestration and lipid production in response to varying growth mode. Bioresour Technol.

[42] Zhao P, Yu X, Li J, Tang X, Huang Z (2014). Enhancing lipid productivity by co-cultivation of *Chlorella* sp. U4341 and *Monoraphidium* sp. FXY-10. J Biosci Bioeng.

[43] Cabanelas ITD, Arbib Z, Chinalia FA, Souza CO, Perales JA, Almeida PF, Druzian JI, Nascimento IA (2013). From waste to energy: microalgae production in wastewater and glycerol. Appl Energy.

[44] Leite GB, Paranjape K, Abdelaziz AEM, Hallenbeck PC (2015). Utilization of biodiesel-derived glycerol or xylose for increased growth and lipid production by indigenous microalgae. Bioresour Technol.

[45] Jiao K, Chang J, Zeng X, Ng I, Xiao Z, Yong S, Xing T, Lu L (2017). 5-Aminolevulinic acid promotes arachidonic acid biosynthesis in the red microalga *Porphyridium purpureum*. Biotechnol Biofuels.

[46] Kavitha MD, Kathiresan S, Bhattacharya S, Sarada R (2016). Culture media optimization of *Porphyridium purpureum*: production potential of biomass, total lipids, arachidonic and eicosapentaenoic acid. J Food Sci Technol.

